# The Role of Tricellular Junctions in the Transport of Macromolecules Across Endothelium

**DOI:** 10.1007/s13239-020-00483-x

**Published:** 2020-08-20

**Authors:** Mean Ghim, Yumnah Mohamied, Peter D. Weinberg

**Affiliations:** 1 Department of Bioengineering, Imperial College London, London, SW7 2AZ UK; 2 Department of Aeronautics, Imperial College London, London, SW7 2AZ UK; 3 Present Address: School of Informatics, University of Edinburgh, Edinburgh, UK; 4 Present Address: Section of Cardiovascular Medicine, Yale Cardiovascular Research Centre, Yale University School of Medicine, New Haven, CT USA

**Keywords:** Endothelial cell, Intercellular cleft, Epithelium, Deep learning, Hemodynamics, Swirling well, FITC-avidin

## Abstract

**Purpose:**

Transport of water and solutes across vascular endothelium is important in normal physiology and critical in the development of various diseases, including atherosclerosis. However, there is debate about the routes for such transport. We recently showed that an albumin-sized tracer crossed endothelium at bicellular and tricellular junctions, a tracer having the size of high density lipoprotein crossed only through tricellular junctions, and a tracer with the size of low density lipoprotein was unable to cross by either route and instead traversed the cells themselves. Here we review previous work on the structure and function of tricellular junctions. We then describe a study in which we assessed the role of such junctions in the transport of an albumin-sized tracer.

**Methods:**

We examined normal endothelial monolayers, the effect of agonists that modify their permeability, and the influence of different patterns of shear stress.

**Results:**

Under normal conditions, approximately 85% of transendothelial transport occurred through tricellular junctions. This fraction was unchanged when permeability was reduced by sphingosine-1-phosphate or increased by thrombin, and also did not differ between endothelium exposed to multidirectional as opposed to uniaxial shear stress despite a > 50% difference in permeability.

**Conclusion:**

These data show that tricellular junctions dominate normal transport of this tracer and largely determine influences of agonists and shear. The effects were attributable to changes in both the number and conductivity of the junctions. Further investigation of these structures will lead to increased understanding of endothelial barrier function and may suggest new therapeutic strategies in disease.

## Introduction

The vascular endothelium functions as a selective barrier between blood and surrounding tissue. It regulates the exchange of water, solutes and cells, maintaining normal homeostasis. Endothelial injury may lead to hyperpermeability, where components of the blood normally confined to the vascular lumen pass through the endothelium, or the rate of such passage is increased. Currently, there are no therapeutics that address this dysfunction, which is a key issue in atherosclerosis, lung injury, vascular leak in cancer, and sepsis, among other diseases. Understanding the mechanisms by which molecules cross the endothelium under both physiological and pathological conditions is therefore crucial.

Here we consider whether tricellular junctions are an important route for the transport of macromolecules across endothelium, and whether they are involved in the physiological and pathological alteration of such transport by agonists and biomechanical forces. This topic has previously received little attention. We start by reviewing what is known about tricellular junctions and then present an experimental study of their role.

## Part I: Review of Transendothelial Transport at Tricellular Junctions

Macromolecules pass through the endothelium either by transcellular or paracellular routes. Transcellular transport refers to solutes and water passing through the endothelial cell itself, from the luminal membrane to the basolateral membrane or in the reverse direction; for lipid-insoluble solutes, it generally involves vesicle-mediated trafficking. The present review concerns paracellular transport, which is the movement of molecules through the space between neighbouring endothelial cells, known as the intercellular cleft or intercellular junction.

Neighbouring cells are connected by protein complexes, also termed junctions, of three types: gap junctions, adherens junctions and tight junctions.^5^ Gap junctions are composed of the connexin protein family, which form intercellular channels linking the cytoplasm of adjacent cells.^37^ They allow for the lateral movement of small mediators between cells, facilitating cell–cell interaction, and are not directly involved in paracellular transport. However, tight junctions and adherens junctions are; together they form a barrier that restricts movement of molecules through the cleft.^3^,^4^,^12^,^36^

Tight junctions are formed by the homotypic binding of three families of transmembrane proteins; occludins, claudins and junctional adhesion molecules A.^2^,^15^,^32^,^33^,^41^ Tight junctions prevent the movement of molecules larger than approximately 2 nm in diameter through the intercellular cleft.^44^ In the blood–brain barrier, they form a continuous seal between neighbouring endothelial cells but elsewhere there are breaks that allow larger molecules through.

Adherens junctions are formed by the interaction of the cadherin family of proteins, with vascular endothelial (VE)-cadherin being the dominant cadherin for vascular endothelial cells.^13^ Unlike tight junctions, adherens junctions allow the passage of molecules up to 20 nm in diameter, which occurs where there are breaks in the tight junction.^44^

Macromolecules > 20 nm in diameter, such as low density lipoprotein (LDL), are thought not to pass through intercellular junctions under normal physiological conditions. However, intercellular junctions appear to become “leaky” when endothelial cells undergo division or death; widening of the cleft will let molecules larger than 20 nm through, and transport of smaller molecules will also be increased. The width of leaky junctions has been estimated to range between 80-1330 nm for mitotic cells and 15–1000 nm for apoptotic cells.^11^ Using a 3-pore model, Cancel *et al*.^8^ calculated that leaky junctions account for 90.9% of LDL transport and 44% of albumin transport through endothelium cultured with a pressure gradient across the monolayer. They subsequently showed that LDL transport can be altered by changing the frequency of apoptosis and proliferation.^9^,^10^

An alternative route for paracellular transport, and one we investigate further in this work, is where three or more cells come together, forming tricellular junctions. In *epithelium*, these junctions are highly specialised and essential in controlling paracellular permeability.^1^,^25^,^30^ The junctional proteins organise to form a channel 10 nm in diameter and 1 *μ*m in length, running from the apical to the basal side of the cell.^19^,^39^ These “central tubes” allow passage of solutes that have a mass of 20 kDa, but not those with a mass of 70 kDa.^24^ Specific proteins in epithelial tricellular junctions have been identified: tricellulin and angulins (LSR/angulin-1, ILDR1/angulin-2 and ILDR2/angulin-3). Both are essential for the structural integrity and formation of tricellular junctions, and knockdown of tricellulin or angulin-1 decreases barrier function.^18^,^29^,^34^ Tricellular junctions are also involved in process such as cell division and cell morphogenesis.^6^,^23^,^35^

**Figure 1:**
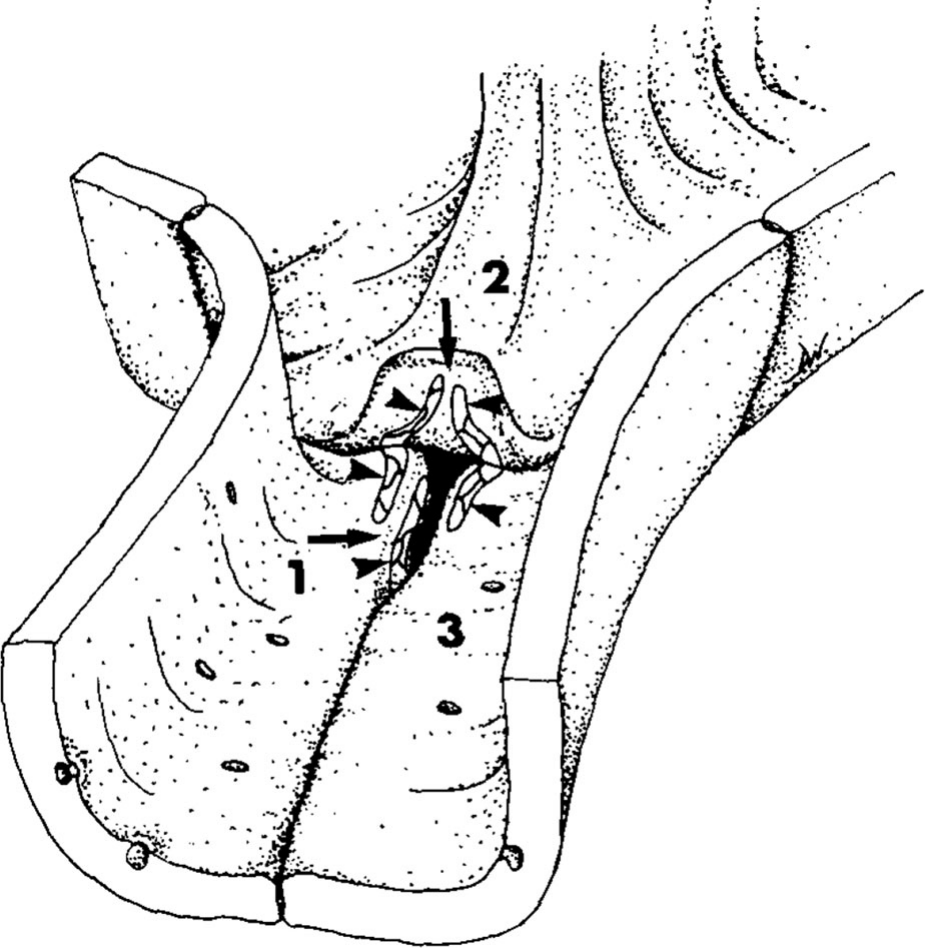
Sketch of an endothelial tricellular junction in a pulmonary capillary, based on freeze fracture electron micrographs. The flap of cell “2” has been peeled back and the gap between cells “1” and “3” has been widened at its end in order to show the tight junction strands (arrowheads). Those on the underside of the flap join with those on the luminal surface of cells “1” and “3”. Those on the edge of cell “1” join with corresponding structures on the edge of cell “3” (which are not shown). Even with the flap lowered and the tight junctions properly formed, a patent channel remains (arrows). Reproduced from Fig. 13 of Walker *et al*. 1994,^45^ Copyright, with permission from Elsevier.

Endothelial tricellular junctions are less well-characterised. An early ultrastructural study of pulmonary capillary endothelium found breaks in tight junction organisation at some tricellular junctions, with average widths of 27 nm, lengths of 1.1 *μ*m and depths ranging from 0.48 to 2.9 *μ*m.^45^ Furthermore, the organisation of the endothelial borders at tricellular junctions differed greatly from epithelial cells: two of the endothelial cells form a bicellular junction with no overlying edges, creating an intercellular cleft, while the third cell, termed the ‘flap’ cell, overlies the bicellular junction (Fig. 1). This organisation suggests a pore-like structure.^45^ It may be that tricellular junctions allow large macromolecules to pass and that they can open and close to control permeability. The ability to close might be lost under pathological conditions.

ICAM-1 enrichment occurs at these junctions and they are the primary sites for leukocyte transmigration.^7^,^42^ Tricellulin and angulin-1 are expressed and localised at tricellular junctions in brain and retinal endothelium.^20^,^28^ Angulin-1 in particular is involved in the formation of the blood–brain barrier: brain endothelium is more permeable to small molecules in angulin-1 knockout mice.^38^ Additionally, angulin-1 expression is reduced in experimental models of stroke and multiple sclerosis, indicating that loss of barrier function at these junctions may be important in the development of disease.^38^

Based on such ultrastructural and functional peculiarities, Tarbell speculated that endothelial tricellular junctions might form part of the large pore system.^43^ We demonstrated that macromolecules do indeed cross cultured endothelial monolayers preferentially at such sites.^16^ We added fluorescein isothiocyanate-labelled avidin (FITC-avidin), which is similar in size to albumin, to the medium above an endothelial monolayer cultured on biotinylated gelatin. In this system, developed by Dubrovskyi *et al*.,^14^ the tracer binds to the substrate on crossing the endothelium; its location can be compared to overlying cellular structures in order to determine its transport route. We found that tricellular junctions were foci of FITC-avidin transport.^16^

Furthermore, we demonstrated that a larger tracer, NeutrAvidin labelled with R-phycoerythrin (a fluorescent protein with a Stokes–Einstein radius of ~ 5.5 nm^27^ and hence similar in size to high density lipoprotein), could cross the monolayer at these intersections even though it could not pass through bicellular junctions.^16^ An even larger tracer—streptavidin labelled with a 20 nm quantum dot, approximately equal in size to low density lipoprotein—could not pass through bicellular or tricelluar junctions.^16^

Despite these advances, many aspects of endothelial tricellular junction structure and function have not been investigated. In Part II, we describe a study of the role of tricellular junctions in macromolecule transport across cultured endothelium and their modification by agonists and biomechanical forces.

## Part II: Experimental Study

### Introduction

In Part I, we made the case for investigating transport through endothelial tricellular junctions, based in part on our previous study which showed that macromolecules travel through such junctions in cultured monolayers.^16^ However, we did not assess the degree to which the transport through the tricellular junctions accounted for overall transport through the monolayer, and thus whether they are the predominant pathway. In the present study, we quantify this for monolayers under normal conditions and after treatment with the known mediators of permeability, sphingosine-1-phosphate (S1P) and thrombin, in order to identify whether the changes in overall transport can be explained by changes in tricellular junctions.

The influence of shear stress on tricellular junctions has also never been investigated. Shear stress is known to alter endothelial permeability^21^,^40^,^46^; it has recently been suggested that increased transport occurs when the endothelium is exposed to multidirectional flow.^16^,^31^ This behaviour may be important in the development of atherosclerosis, which is dependent on the transendothelial transport of blood-borne, cholesterol-carrying macromolecules into and out of the arterial wall and, it has been suggested, on multidirectional flow.^31^ How permeability is increased by shear stress is still under debate. Here we investigate the permeability of tricellular junctions, and of the endothelium as a whole, under both uniaxial (putatively atheroprotective) and multidirectional (putatively atherogenic) shear.

A challenge in any study involving a spatially-resolved analysis of tricellular junctions is actually locating the junctions in sufficient numbers: hand annotation is so arduous that it poses limitations on the quantity of data that can be processed, and hence on reliability. We use semantic segmentation technology based on machine learning—specifically, deep neural network models—to identify tricellular junctions automatically.

### Methods

#### Cell Culture

Human aortic endothelial cells (HAECs; PromoCell) were cultured in Endothelial Cell Growth Medium MV (ECGM; PromoCell) with 5% (vol/vol) fetal bovine serum (FBS), 4 *μ*L/mL endothelial cell growth supplement, 90 *μ*g/mL heparin and 1*μ*g/mL hydrocortisone. All experiments were carried out using HAECs of passage 5.

#### Application of Shear Stress

HAECs were seeded in 12-well plates coated with biotinylated-gelatin, prepared using the methods of Dubrovskyi *et al*.^14^ Once the cells were confluent, the plate was placed on an orbital shaker (PSU-10i, Grant Instruments) and swirled for 72 h. The depth of medium within the wells when they were stationary was 2 mm. The shaker platform had a rotational rate of 150 rpm and a circular orbit with a diameter of 10 mm in the horizontal plane. This system gives multidirectional flow in the centre of the well and uniaxial flow at the edge.^16^

#### Application of Tracer

Medium was replaced with reduced-serum ECGM (2.5% FBS). After 24 h on the shaker (or stationary for controls), FITC-avidin (final concentration 0.38 *μ*M) was added to this medium and the wells were returned to the incubator, on or off the shaker as required, for a further 3 min. The tracer solution was then removed, and the wells were rinsed 3x with PBS and fixed with 4% paraformaldehyde for 10 min.

To modify permeability, confluent HAEC monolayers were treated with reduced-serum ECGM containing 1*μ*M of S1P (Cayman Chemicals) for 1 h or 1 U/mL of thrombin (Sigma-Aldrich) for 30 min, prior to FITC-avidin application.

#### Immunofluorescent Staining for Cell Borders

Fixed HAECs were permeabilised and blocked at room temperature with 0.1% Triton X and 2% bovine serum albumin for 1 h. They were then incubated with a 1:300 dilution of mouse anti-human VE-cadherin (BD Pharmingen) overnight at 4 °C followed by 1 h with Alexa Flour 568 goat anti-mouse (Thermo Fisher Scientific) at room temperature.

#### Image Acquisition

A Leica SP5 inverted confocal microscope with a × 10, 0.40 NA objective and × 2 zoom was used to image monolayers. FITC-avidin and Alex Fluor 568 were excited at 488 and 561 nm and detected at 500–525 and 590–625 nm, respectively. Five images were obtained of each static well in the S1P and thrombin experiments, one image at the centre of the well and one each at the 12, 3, 6 and 9 o’clock positions, 4 mm away from the wall of the well. For shear experiments, nine images were obtained at the centre of the well in a 3 × 3 grid, and a further nine images towards the edge of the well, 8.5 mm from the centre of the well.

For live cell imaging of confluent HAECs cultured under static conditions, medium was replaced with ECGM supplemented with 20 mM HEPES and placed in a pre-warmed 37 °C temperature-controlled imaging chamber. The tracer solution (made up in ECGM supplemented with 20 mM HEPES) was added 9 s after imaging commenced, and the cells were imaged for approximately 12 min.

#### Semantic Segmentation of Tricellular Junctions Using Machine Learning

Semantic segmentation is the task of classifying each individual pixel in an image and assigning it a label. The RefineNet architecture proposed by Lin *et al*.^26^ was used to classify each pixel as either a tricellular junction or not. The RefineNet architecture consists of stacks of convolutional layers followed by ReLU activation layers with residual connections, which extract features in the input image at multiple resolutions and subsequently fuse them to generate a high-resolution prediction of the pixel labels.

Data to train the model consisted of grayscale images of cells with immunostained borders obtained as described above, and corresponding grayscale images of segmented tricellular junctions obtained by hand annotation. A total of twelve images, sized 1024 × 1024 pixels, of cells stained with anti-VE-cadherin were obtained, at both the centre and towards the edge of the well and under both static and sheared conditions. To achieve data augmentation for training, ten of the twelve images were cropped into smaller images, rotated by 90, 180 and 270 degrees and horizontally flipped. Further data augmentation was carried out during training by random colour jittering. The remaining two of the original twelve images were retained for testing. In total, training used approximately 12,100 individual tricellular junctions and testing used 5700.

An open-source Pytorch implementation of RefineNet was used (https://github.com/thomasjpfan/pytorch_refinenet) and the model was tuned using a subset of the training data as a validation dataset (training on 1024 images, validating on 256). The final model was trained on input images of size 256 × 256 pixels, batch size of 32, learning rate of 0.00001, convolutional filter size of 3, stride of 1 and channel number of 128, using the Adam optimiser (weight decay 0) for 320 epochs (loops through the entire dataset), corresponding to approximately 12,000 training updates.

Far fewer pixels were labelled as a tricellular junction than not. Hence using a pixel-wise cross entropy loss as the training objective would have led to training being dominated by regions of cells that were not tricellular junctions. The soft Dice coefficient was therefore used; it is a measure of overlap between two images. The model was trained to minimise this value.

The output from the trained model was processed in MATLAB. The watershed transform was used to generate a binary map, which was necessary to split contiguous regions where two tricellular junctions appeared as one in the unprocessed predicted output. The centroid of each tricellular junction was used to identify whether a region of increased tracer accumulation was close enough to a tricellular junction for that junction to be classified as being *permeable*.

To differentiate tracer accumulation from background fluorescence, images of bound FITC-avidin were subject to MATLAB-based adaptive thresholding. Total tracer accumulation was calculated as the sum of all pixel values in the thresholded image. Spots of tracer accumulation (defined by having connected components) were each given a label. In conjunction with the semantic segmentation, described above, this permitted the identification of *permeable* tricellular junctions and the tracer transport associated with them, using a distance threshold from the centre of a tricellular junction to the closest pixel of a high-permeability spot of 2 pixels.

This analysis gave the ratio of permeable tricellular junctions to all tricellular junctions, the total tracer transport through the monolayer, the ratio of tracer through tricellular junctions to total transport and, lastly, an estimate of the average tracer transport through each permeable tricellular junction.

#### Statistical Analysis

Statistical significance was assessed by Student’s unpaired *t* test using *p* < 0.05 as the criterion for significance. *, **, and *** denoted *p* < 0.05, *p* < 0.01, *p* < 0.001, respectively.

### Results

#### Machine Learning Predictions of Tricellular Junctions

**Figure 2:**
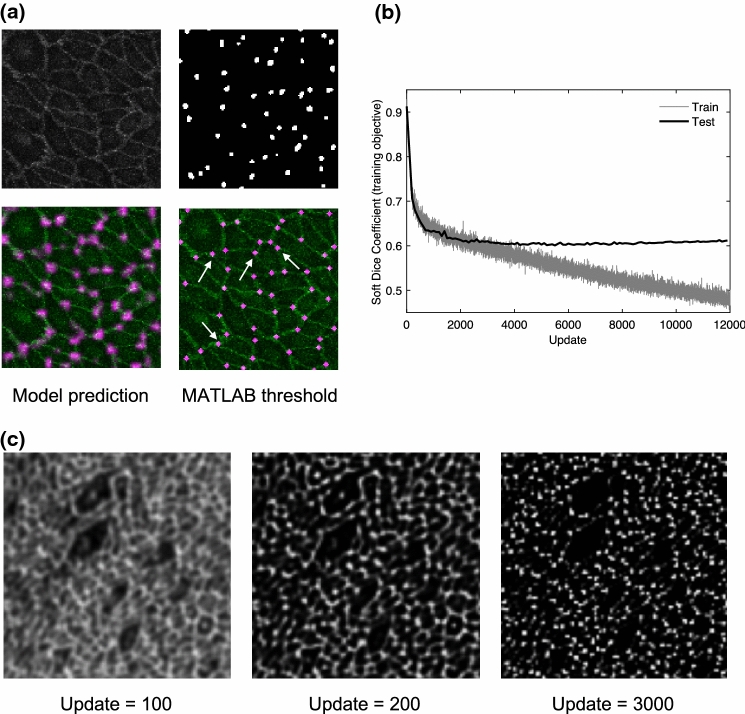
(a) Top row shows a region of a test image of VE-cadherin stained cell borders (left) and hand annotated labels of tricellular junctions (right); these are the inputs to the machine learning model. Bottom row shows model predicted output overlaid with cell borders (left) and the thresholded binary map generated with MATLAB (right). The white arrows point to junctions correctly predicted that were missed and falsely labelled by the human annotator. (b) Soft Dice coefficient (the loss function) as a function of the number of training updates, for training datasets and test datasets. (c) Example model prediction of tricellular junctions on a test image at three points during training.

The top row of Fig. 2a shows a region of a test image (left) with immunostained cell borders, and hand-annotated tricellular junctions (right). The model was trained on pairs of data such as this. The bottom row shows the prediction by the model of the location of tricellular junctions, overlain with the cell borders (left), and the thresholded binary map generated in MATLAB, assuming a uniform size of tricellular junction (right). The intensity of the purple spots in the output reflects the certainty of the model that the region is a tricellular junction. Interestingly, there were examples of junctions that were correctly identified by the trained model but not by the human annotator (indicated by white arrows).

Figure 2b shows the evolution towards the training objective. As expected, the loss function (the measure of dissimilarity between the true label and the prediction) decreased for the training data as training progressed; however, for the test data, it reached a minimum at around update 3,000, and then slightly increased as training progressed further. This is symptomatic of overfitting, which is when the model begins to memorise the training data, rather than learn meaningful information from it.

Figure 2c shows examples of the model prediction of test images during training. From Fig. 2b, it is clear that most learning occurs in the first few hundred training updates; hence predictions are shown for 100, 200 and 3000 updates. The model rapidly learned to identify cell borders, and after a longer time it learned to discard the bicellular junctions and label only tricellular junctions.

Using Fig. 2b, and qualitatively assessing the model predictions over the test images (Fig. 2c), update 3,000 was selected as the final trained model.

#### Visualisation of Tracer Accumulation Using Live Cells and Real-Time Acquisition

**Figure 3:**
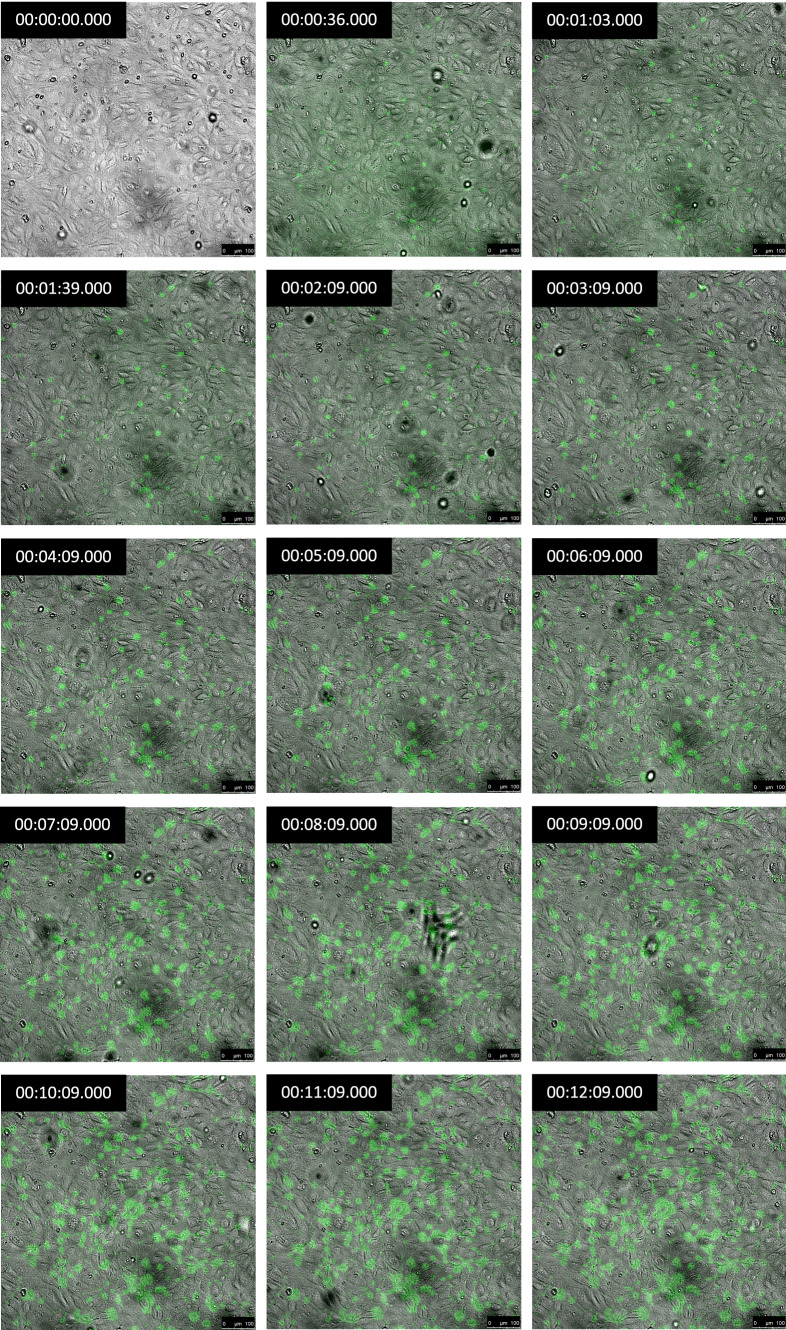
Live-cell acquisition of FITC-avidin accumulation (green) under an HAEC monolayer (phase contrast) from 0 to 12 min after the start of imaging. Tracer was added at 9 s. Time stamps are in the top left corner of each panel. Bar = 100 *μ*m.

Continuous live-cell imaging of tracer transport revealed a gradual increase in FITC-avidin accumulation over time; spots of high uptake were evident and the size and number of the spots both increased (Fig. 3).

#### Effect on Tracer Accumulation of Treatment with S1P and Thrombin

**Figure 4:**
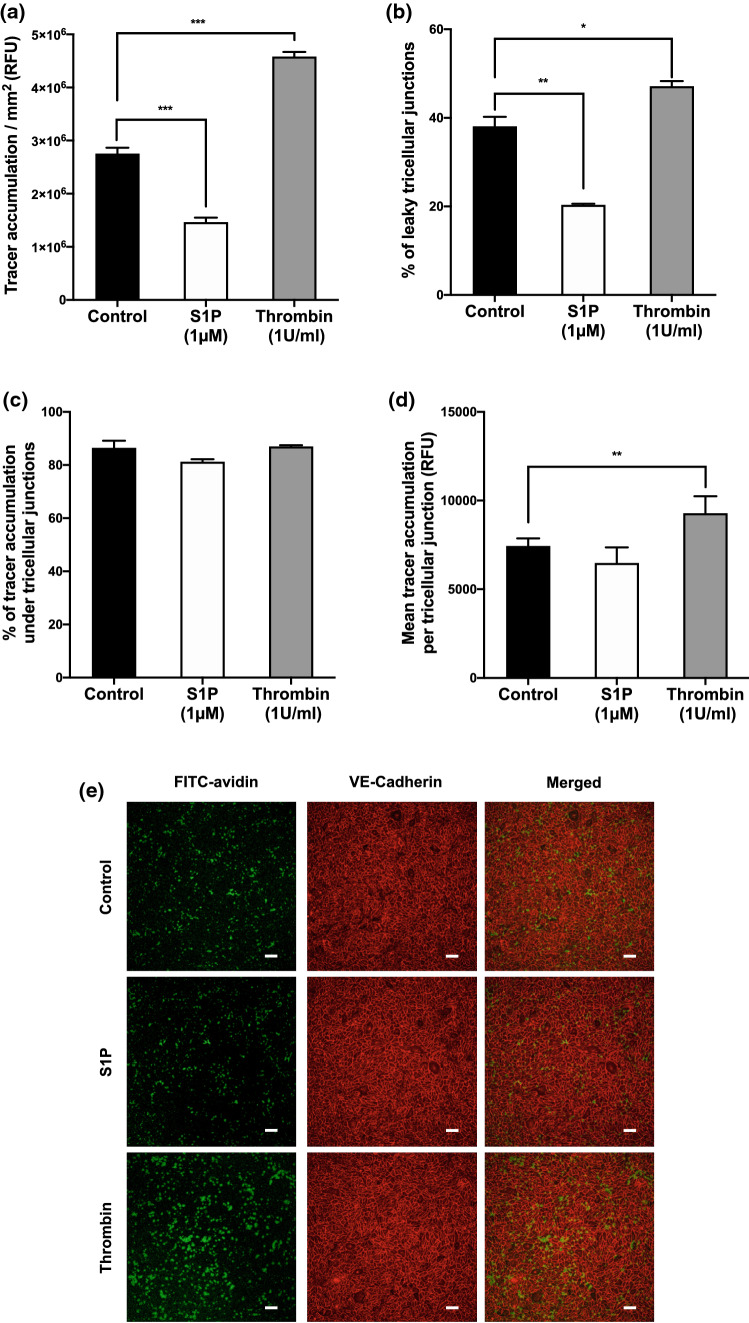
(a) Total FITC-avidin accumulation, (b) percentage of permeable tricellular junctions, (c) percentage of the total tracer accumulation due to tricellular junctions, (d) mean tracer accumulation per permeable tricellular junction, and (e) confocal images (tracer accumulation, green; VE-cadherin, red), all for untreated HAEC monolayers or monolayers treated with S1P or thrombin. *RFU* relative fluorescence units. Bar = 100 *μ*m.

Overall tracer accumulation was decreased 46% by S1P treatment and increased 40% by thrombin treatment, compared to untreated monolayers (Fig. 4a; *p* < 0.001 for both, *n* = 3 donor aortas). In untreated monolayers, 38.1 ± 2.1% (mean ± SEM) of all tricellular junctions were permeable. This decreased to 20.3 ± 0.3% in S1P-treated monolayers, and increased to 47.3 ± 1.8% in thrombin-treated monolayers (Fig. 4b; *p* < 0.01, *p* < 0.05). Tricellular junctions accounted for > 80% of the overall tracer accumulation of the monolayers for all three conditions, and there was no significant difference between conditions (Fig. 4c; *p* > 0.05 for both). The mean tracer accumulation per permeable tricellular junction was increased ~ 25% for monolayers treated with thrombin compared to untreated monolayers; no significant difference was seen between untreated monolayers and those treated with S1P (Fig. 4d; *p* < 0.01, *p* > 0.05).

#### Tracer Accumulation Under Shear

**Figure 5:**
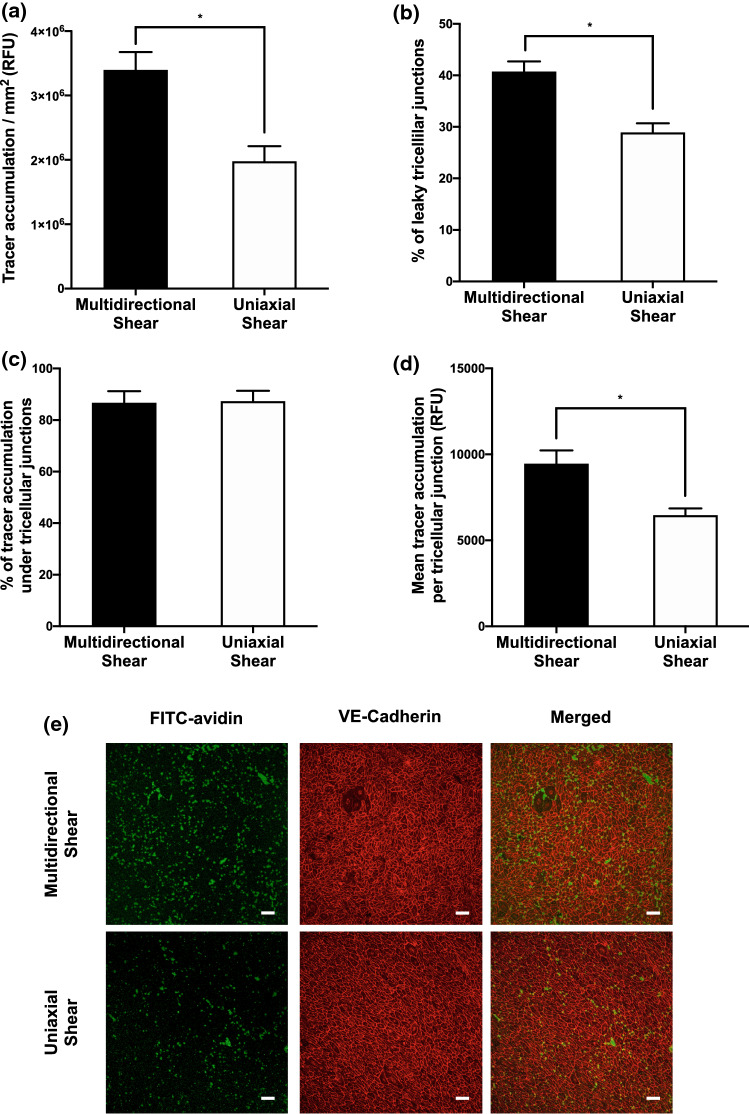
(a) Total FITC-avidin accumulation, (b) Percentage of permeable tricellular junctions, (c) percentage of the total tracer accumulation due to tricellular junctions, (d) mean tracer accumulation per permeable tricellular junction, and (e) confocal images (tracer accumulation, green; VE-cadherin, red) all for HAEC monolayers exposed to multidirectional or uniaxial shear stress. *RFU* relative fluorescence units. Bar = 100 *μ*m.

Tracer accumulation under HAECs exposed to approximately uniaxial shear stress was ~ 40% lower than under HAECs exposed to multidirectional shear stress (Fig. 5a; *p* < 0.05). For multidirectionally sheared cells, 40.7 ± 2.0% (mean ± SEM, *n* = 3 donor aortas) of tricellular junctions were found to be permeable and these permeable junctions accounted for 85% of total tracer accumulation (Figs. 5b and 5c). For cells experiencing uniaxial flow, fewer tricellular junctions were found to be permeable (28.9 ± 1.8%), and these similarly accounted for 87% of the total tracer accumulation (Figs. 5b and 5c; *p* < 0.05, *p* > 0.05). The mean tracer accumulation per permeable tricellular junction for monolayers exposed to uniaxial shear also saw a decrease, by ~ 31% compared to cells which were subject to multidirectional flow (Fig. 5d; *p* < 0.05).

### Discussion

In this study we investigated whether endothelial tricellular junctions play a critical role in paracellular permeability and its regulation by agonists and biomechanical forces, using a recently developed, spatially resolved permeability assay. We also employed state-of-the-art deep learning techniques to analyse the data. More specifically, we made use of a newly proposed architecture in the field of supervised semantic segmentation (RefineNet) and demonstrated the ease with which the method can be applied, given sufficient training data. Not only did it enable the analysis of large quantities of data (approximately 13,200 tricellular junctions were identified in this study), the method also outperformed manual labelling: some tricellular junctions were correctly labelled by the trained machine but missed by the human annotator (Fig. 2).

Live-cell imaging revealed spots of elevated subcellular accumulation of FITC-avidin which appeared to occur where three or more endothelial cell borders came together (Fig. 3). That is consistent with tricellular junctions being important routes for paracellular transport. The size of the spots tended to increase with time (Fig. 3), suggesting that the tricellular junctions above them remained open throughout the imaging and that the tracer was diffusing further in the lateral direction before being arrested through binding to biotin. (The increase in spot radius is too small to be due to lateral diffusion *without* binding: that would give a radius of ~ 350 *μ*m after 12 min.) The number of tracer spots near tricellular junctions also increased with time (Fig. 3), suggesting that some tricellular junctions provide more resistance to the movement of the tracer than others and lead to detectable tracer accumulation only in the later images. An alternative explanation is the opening of tricellular junctions that had previously blocked transport of tracer. The dynamics of tricellular junctions could be investigated further by using tracer labelled with different fluorescent dyes, added at different times.

The significance of tricellular junctions in the subendothelial accumulation of FITC-avidin was quantified: tricellular junctions accounted for > 80% of total transport through the monolayer (Fig. 4c). This transport was associated with less than 40% of tricellular junctions under control conditions. Both trends are easily visible in the merged channel of Fig. 4e. Given the results discussed in the previous paragraph, we speculate that had accumulation over a shorter time been measured, an even lower proportion of the junctions would have been sufficiently permeable to show detectable transport. Furthermore, if transport through other routes is slower, then tricellular junctions would have accounted for an even larger fraction of the total transport. (Bicellular junctions appeared somewhat less involved in the present study than in our earlier work,^16^ which may be due to the use of human rather than porcine aortic endothelial cells and/or the use of endothelial-specific culture medium.)

Endothelial permeability to macromolecules can be decreased or increased by agonists. We imaged the permeability-reducing effect of S1P^47^ and the permeability-increasing effect of thrombin^22^ in order to determine the involvement of tricellular junctions. We found, consistent with the earlier results for other molecules, that overall transport of FITC-avidin was decreased by S1P and increased by thrombin (Fig. 4a). The fraction of tracer transport occurring *via* tricellular junctions remained constant across conditions at 80-85% (Fig. 4c). This finding demonstrates the importance of tricellular junctions in regulating paracellular permeability; they accounted for the majority of the changes. Since the fraction of transport occurring by other routes also remained approximately constant, the results are consistent with a common regulatory effect of the agonists on tricellular and bicellular junctions.

Either the number of permeable tricellular junctions or their resistance, or both, must have been modified. Analysis showed that the effect of S1P was almost entirely due to a reduction in the number of permeable tricellular junctions, whereas the effect of thrombin could be attributed to increases in both their number and conductivity (Figs. 4b and 4d).

Mechanical forces, and in particular haemodynamic wall shear stress, also modify endothelial permeability. For example, when tissue culture wells containing endothelial monolayers are swirled on an orbital shaker, paracellular permeability increases in the centre of the well, where flow is multidirectional, and decreases towards the edge of the well, where flow is more uniaxial.^16^ We determined the role of tricelluar junctions in this behaviour. As in our earlier study, tracer accumulation was higher under cells exposed to multidirectional shear than under those exposed to uniaxial shear (Fig. 5a). The fraction of tracer transport occurring through tricellular junctions was again constant across conditions at around 85% (Fig. 5c), indicating a difference in transport through the tricelluar junctions, and through other routes, between the two conditions; the effect of changes in tricellular junctions determined the majority of the effect of shear. As with thrombin, it was attributable to changes in both the number of permeable tricellular junctions and the conductivity of each one (Figs. 5b and 5d).

The routes by which macromolecules cross endothelium have been debated for decades. Grotte *et al*.^17^ observed that the lymph-to-plasma concentration ratio of circulating dextrans decreased abruptly when their diameter was increased beyond ~ 4 nm, but only minor decreases in the ratio were observed with further increases in diameter. From these observations, Grotte proposed the “two-pore theory”, where one pore allowed the passage of molecules smaller than ~ 4 nm and a rarer but much bigger pore allowed the passage of molecules larger than ~ 4 nm.

Using the spatially resolved permeability assay and avidin-based tracers of different size, we previously identified three “pores” – bicellular junctions, tricellular junctions and transcellular transport. (These are different to the three pores identified by the the Tarbell group.^8^) As noted above, tricellular junctions permitted passage of a tracer as large as high density lipoprotein, which was not able to pass through bicellular junctions. A tracer similar in size to low density lipoprotein could not pass through either route; it was transported cross the cells themselves. Here we have confirmed that tricellular junctions are an important route for transendothelial transport and have further shown that they can be the main determinant of changes in transport caused by agonists or mechanical forces.

Our data may be relevant to the development of disease, as well as to normal physiology. In particular, atherosclerosis is characterised by the subendothelial accumulation of cholesterol and other lipids; the primary carrier of cholesterol into the subendothelial space is low density lipoprotein, whilst high density lipoprotein is responsible for reverse cholesterol transport. The development of atherosclerosis depends on biomechanical forces and although the nature of these forces remains controversial, some studies suggest that lesions have a predilection for areas of arteries which experience multidirectional flow.^31^

A limitation of the present study is that paracellular permeability is higher *in vitro* than *in vivo*; the underlying transport routes may therefore also be different. A further limitation is that there was no pressure difference across the monolayer and hence no convection; that may have altered the structure of the junctions as well as the mechanism of transport through them, compared to the situation *in vivo*. We previously demonstrated an *in vivo* analogue of the technique used in the present study. The method involves conjugating fluorescent dyes to antibodies raised against components of the basement membrane; the labelled antibodies provide a tracer that can be introduced into the circulation and that will be immobilised on crossing the endothelium. Our *en face* image of the aorta of a rabbit which was administered rhodamine-labelled IgG raised against the non-helical domain of collagen type IV does show spots of enhanced uptake at tricellular junctions (Fig. 10 in Ghim *et al*.^16^), consistent with the present *in vitro* data.

In conclusion, we have reviewed the literature concerning endothelial tricellular junctions and provided experimental evidence that such junctions play a key role in regulating permeability to macromolecules under both normal and potentially pathological conditions. Further investigation of tricellular junctional structure and its regulation may provide new insights into endothelial barrier function and could suggest novel ways to combat diseases related to endothelial permeability.
